# Enzyme-Linked Immunosorbent Assay (ELISA) Development for Equine Serum Amyloid A (SAA) Determination Using Recombinant Proteins

**DOI:** 10.3390/mps8020037

**Published:** 2025-04-07

**Authors:** Pollyanna C. Souto, Marcus R. Santos, Andrés M. Ortega Orozco, Lucas D. Bento, Camilo J. Ramirez-Lopez, Fabrícia M. Girardi, Júlia C. Assis Machado, Leandro L. de Oliveira, Leandro A. da Fonseca

**Affiliations:** 1 Laboratory of Veterinary Clinical Pathology, Department of Veterinary Medicine, Universidade Federal de Viçosa, Viçosa 36570-900, Brazil; pollyannasouto@hotmail.com (P.C.S.); amauricioortega@gmail.com (A.M.O.O.); lucasdrumond29@gmail.com (L.D.B.); fabriciamgirardi@gmail.com (F.M.G.); julia.machado@ufv.br (J.C.A.M.); 2 Laboratory of Immunobiologics and Bacteriosis, Department of Veterinary Medicine, Universidade Federal de Viçosa, Viçosa 36570-900, Brazil; marcusrsantos@yahoo.com.br; 3 Laboratory of Animal Reproduction, Department of Veterinary Medicine, Universidade Federal de Viçosa, Viçosa 36570-900, Brazil; camilo.lopez@ufv.br; 4 Laboratory of Immunology and Glycobiology, Department of Biology, Universidade Federal de Viçosa, Viçosa 36570-900, Brazil; leandro.licursi@ufv.br

**Keywords:** protein, acute-phase, inflammation, horse, immunoassay

## Abstract

We aimed to develop a species-specific ELISA for qualitatively and quantitatively determining serum amyloid A (SAA) in horses. Current methods for measuring SAA in horses utilize ELISA or immunoturbidimetric tests designed for human SAA, which are not specific to horses. Mice and rabbits were used to generate polyclonal antibodies against equine SAA. The study examined serum samples from 32 horses with acute inflammatory disease (SG) and 25 clinically healthy horses. Furthermore, the SAAeq kinetics were observed in three horses from the SG group at three different timepoints. The SAA-ELISA established a cut-off at 0.06 OD_492_nm, where values equal to or higher than this were deemed positive, while values below it was considered negative. The test exhibited a sensitivity of 94% and specificity of 92%, resulting in an overall accuracy of 93%. The positive and negative predictive values were 94% and 92%, respectively. Coefficients of variation for inter- and intra-assay were 6.1% and 7.46% for SG and 9.6% and 9.63% for the control group (CG). The detection limit was determined to be 0.067. The SAA-ELISA proved its worth by demonstrating satisfactory performance, paving the way for the development of automated quantitative tests and species-specific semi-quantitative tests. This paves the way for their application in practical field settings.

## 1. Introduction

Acute-phase proteins (APPs) constitute components of the innate immune response, displaying a non-specific nature. Their concentrations directly correlate with the severity of tissue damage [1], rendering them valuable quantitative markers for diagnosis, prognosis, and monitoring instituted therapy [2]. Among these proteins, serum amyloid A (SAA) stands out as one of the most sensitive, both in humans and animals [3], and is particularly significant in horses [4].

Serum amyloid A has emerged as a crucial biomarker in equine medicine, particularly for the diagnosis and monitoring of inflammatory conditions [5]. Its rapid response to inflammatory stimuli allows for the assessment of inflammation severity and the monitoring of patients’ clinical progress. Studies indicate that SAA can be used at various stages of clinical management, aiding in the early detection of inflammatory diseases, prognosis determination, and treatment response monitoring [6]. Furthermore, its application in equine sports medicine has enabled the identification of subclinical pathologies, contributing to the performance management of horses [1,5]. Compared to other acute-phase proteins, such as fibrinogen and haptoglobin, SAA exhibits a more rapid and pronounced response to inflammatory stimuli, making it a superior marker for real-time monitoring of systemic inflammation [7,8]. Elevated SAA levels have been observed in various conditions, including surgical procedures, joint disease, sepsis, pneumonia, diarrhea [9,10], prolonged exercise [11], soft tissue trauma, extensive wounds, intra-articular infection, and colic with primary inflammatory cause (peritonitis, enteritis, colitis, or abdominal abscess) [12,13,14]. This sensitivity makes SAA a useful tool for establishing the prognosis of these diseases [15].

Previous studies have demonstrated that SAA levels rise significantly in response to infectious and non-infectious inflammatory conditions in horses, making it a reliable biomarker for systemic inflammation. However, discrepancies in measurement techniques have been observed, with some assays showing variations in sensitivity and specificity depending on the inflammatory stimulus and sample handling conditions [16,17,18].

Quantifying serum amyloid A (SAAeq) poses a current challenge as there is no established gold standard method for this purpose. Although validated assays for measuring SAA in humans have been adapted for equine SAA (SAAeq), such as the LZ-SAA and VET-SAA immunochromatographic tests (Eiken Chemical) [19,20] and the POC-Stablelab (Epona Biotech) [21], these tests exhibit notable differences in performance and application when used in horses. The LZ-SAA shows a significant correlation with other methods; however, it may present imprecision and a hook effect at high concentrations, making it beneficial for monitoring systemic inflammation [4]. In contrast, the VET-SAA has a broad working range, demonstrating good precision and accuracy, and can detect low SAA levels. However, it is not suitable for extremely high concentrations; despite this, it is widely used in equine practice [20].

Commercial equine-specific SAA kits are available on the market; however, there is no universally accepted gold standard for equine SAA quantification. Differences in assay performance, including detection limits and cross-reactivity with non-equine SAA isoforms, may contribute to discrepancies in reported concentrations across studies [3,22]. The lack of a standard method complicates the establishment of reference values and the direct comparison of results across different laboratories, making standardization a key challenge in equine SAA quantification. Although commercial kits provide a practical alternative, their variability in accuracy and specificity raises concerns regarding their consistency across different settings and clinical conditions. Consequently, despite the existence of commercial tests, their accuracy and reliability remain variable, reinforcing the need for species-specific assay development. Additionally, in Brazil, this technology is not yet widely available, rendering the use of these tests costly.

Hence, the primary objective of this study was to develop and standardize an enzyme-linked immunosorbent assay (ELISA) for the qualitative and quantitative determination of serum amyloid A (SAA) in the equine species.

## 2. Materials and Methods

### 2.1. Recombinant SAA Expression

Equine serum amyloid A (SAA1_horse) was constructed and cloned into the pET-14b plasmid (Biomatik, Kitchener, ON, Canada) for expression in competent *E. coli* cells. The plasmid was transformed into the cells and incubated at 37 °C in Luria–Bertani (LB) medium. The transformed cells were plated on LB agar supplemented with ampicillin and chloramphenicol (100 mg/L) and incubated at 37 °C for 12 h. The resulting colonies were collected and incubated in LB until the desired optical density (OD_600_) was reached. Induction was performed with different concentrations of isopropyl-β-D-1-thiogalactopyranoside (IPTG) (0.4, 0.8, and 1 mM) and incubations were carried out for either 4 h at 37 °C or 16 h at 20 °C.

Following centrifugation, the bacterial pellet was suspended in lysis buffer (50 mM NaHCO_3_, 60 mM NaCl, pH 7.5) and sonicated (six cycles of 10 s at 300 W, with 10-s intervals) to obtain the soluble fraction. The insoluble fraction was solubilized in lysis buffer supplemented with 8 M urea and 20 mM imidazole. Protein expression was evaluated by 15% SDS-PAGE and purification was performed using a HisTrap column followed by dialysis with semipermeable membranes with 35 mm pores (12 kDa cut-off) under different urea gradients (3, 1, and 0 M) at 4 °C.

### 2.2. Characterization of the Protein by Western Blotting

Protein separation was carried out by running a 15% SDS-PAGE gel, followed by transfer onto a nitrocellulose membrane. The membrane was blocked with a 3% PBSBSA solution (PBS and 3% bovine serum albumin) for 14 h at room temperature. After two washes with 0.05% PBST (PBS and 0.05% Tween 20), the membrane was incubated with a mouse anti-His antibody (Sigma-Aldrich, Burlington, NJ, USA) at a 1:2000 dilution in 1% PBSBSA for 2 h at room temperature.

Subsequently, five washes were performed before incubation with a mouse anti-IgG antibody (Sigma-Aldrich, Burlington, NJ, USA) at a 1:5000 dilution in 1% PBSBSA for another 2 h. After an additional five washes, the immunoreactive band of the protein was revealed using a PBSDAB solution (PBS, 3,3 tetrahydrochloride DAB, and 0.015% hydrogen peroxide).

### 2.3. Animal Immunization

Two young adult rabbits were immunized with 100 µg of recombinant equine serum amyloid A (rSAAeq) antigens in complete Freund’s adjuvant (Sigma-Aldrich, Burlington, NJ, USA) at a 1:1 ratio, following the protocol described in [10]. The immunization process was repeated on days 15, 30, and 45 post-initial inoculation. On the 55th day, an intramuscular injection of an anesthetic and muscle relaxant combination was administered, and hyperimmune serum was collected via the external jugular vein.

Simultaneously, twenty 6-week-old male BALB/c mice were immunized with 20 µg of recombinant antigens through intraperitoneal injection, emulsified in complete Freund’s adjuvant (Sigma-Aldrich, Burlington, NJ, USA) at a 1:1 ratio. The procedure was repeated on days 15, 30, and 45 post-initial inoculation. On the 55th day, total blood collection was performed via intraocular puncture. The sera from both rabbits and mice were individually diluted in deionized H_2_O q.s.p., filtered, and treated with ammonium sulfate solution (pH 6.0 at 45%) to precipitate immunoglobulins. After 30 min of incubation on ice, the solutions were centrifuged, the supernatant was discarded, and the globulins were resuspended in PBS. The resulting solutions were stored at 6 °C for protein measurement.

### 2.4. Optimization of Antibody Concentrations

To determine the optimal working concentrations of the antibodies, serial dilution experiments were performed. The capture antibody (anti-SAAeq1) was tested at dilutions of 1:10, 1:20, 1:50, 1:80, and 1:100, while the detection antibody (anti-SAAeq2) was tested at dilutions of 1:10, 1:20, 1:50, and 1:80. The ideal concentrations were selected based on the highest signal-to-noise ratio and the best linearity of the standard curve, ensuring a coefficient of determination (R^2^) close to 1. The best performance was achieved with a 1:100 dilution for anti-SAAeq1 and a 1:80 dilution for anti-SAAeq2, as these concentrations provided a high correlation coefficient (R^2^ = 0.9995), optimal absorbance values, and minimal background noise.

### 2.5. ELISA Assay

Polyclonal antibodies against SAAeq produced in rabbits (anti-SAAeq1) and mice (anti-SAAeq2) were used in an ELISA assay. Similarly, mouse anti-IgG conjugated with peroxidase (anti-IgGca3) (Sigma Aldrich, Burlington, NJ, USA) was also employed. Microplates (Nunc Immuno 96 MicroWell—Plates MaxiSorp, Thermo Fisher Scientific, Waltham, MA, USA) were sensitized with 100 µL of anti-SAAeq1 at the optimized dilution of 1:100 in 0.05 M carbonate buffer (pH 9.6) by overnight incubation at 4 °C. After three washes with PBST, the plates were blocked with 100 µL of 3% PBSBSA for 1 h with shaking at room temperature. Plate washes were repeated between each incubation and constant incubation conditions were maintained throughout the process, varying only the duration.

Following this, 100 µL of diluted SAAr in 1% PBSBSA was added and the plates were incubated for 1 h again. Subsequently, the plates were incubated with 100 µL of diluted anti-SAAeq2 at the optimized dilution of 1:80 in 1% PBSBSA for 2 h. Finally, 100 µL of diluted anti-IgGca3 in 1% PBSBSA was added. To stop the reaction, 30 µL of 3N H_2_SO_4_ solution was used and the absorbance reading was taken at 492 nm. The rSAAeq was incubated in eight dilutions (1:2) ranging from 150 mg/L to 1.17 mg/L.

With the ideal antibody concentration determined, field samples were evaluated at dilutions of 1:10, 1:50, and 1:100, tested in triplicate. Three serum samples from horses with acute inflammatory processes containing the SAA antigen (identified by mass spectrometry-MALDI/TOF-TOF) and three samples from healthy animals without any inflammatory process were employed to determine the ideal working concentration.

### 2.6. Animals and Samples

Approval for this study was obtained from the Ethics Committee on Animal Use of the Federal University of Viçosa (UFV), under protocol number 45/2019.

Samples were received from the Veterinary Hospital of UFV (HOVET-UFV) and the Equine Section of the Department of Animal Science of the Federal University of Viçosa (DZO-UFV). All horses included in the study belonged to the Mangalarga Marchador breed. The control group (CG) consisted of 25 healthy horses with a mean age of 7.9 years. The sick group (SG) included 32 horses diagnosed with acute inflammatory disease, with a mean age of 3.1 years. To assess the assay’s ability to detect systemic inflammation, serum samples from both groups were analyzed. Within the SG, 13 horses presented acute inflammatory disease associated with at least one clinical alteration detected by laboratory tests or physical examination. Additionally, 19 horses exhibited acute inflammatory conditions (colic, endotoxemia, dental disease, or recent surgical intervention) associated with at least two clinical alterations and the presence of the SAAeq protein, confirmed by mass spectrometry (MALDI/TOF-TOF). The correlation between specific diagnoses and respective SAA levels was assessed to determine the biomarker’s effectiveness in identifying systemic inflammation.

To evaluate the kinetics of the SAAeq protein, three individual animals from the sick group (SG) with various clinical conditions (dental fistula, hemoparasitosis, and penile laceration) were selected and monitored at three distinct time points: D0 (arrival at the hospital), D3 (3 days after starting treatment), and D7 (7 days after starting treatment).

### 2.7. Assay Characteristics

The cut-off point that distinguishes positive from negative reactions was defined based on the average absorbance values of negative samples plus twice the standard deviation (SD) of those samples. The standard deviation value used in the formula ensured that the confidence level of the result was 7. In other words, for a 95% confidence level, the cut-off point was calculated as μ OD + 2x SD. Consequently, samples with an OD_492_ nm greater than the cut-off were considered positive.

To determine the diagnostic sensitivity (SD) and specificity (ED) of the ELISA test, the following formulas were used: SD = TP/(TP + FN) × 100, which calculates the percentage of truly diseased individuals (TP) correctly identified by the test out of all individuals with the disease (TP + FN); and ED = TN/(TP + TN) × 100, which calculates the percentage of truly healthy individuals (TN) correctly identified by the test out of all healthy individuals (TN + FP).

Accuracy was calculated using the formula: accuracy = (TP + TN)/total number of serum samples tested × 100, which measures the overall performance of the test by considering both sensitivity and specificity. Furthermore, the positive predictive value (PPV) and negative predictive value (NPV) were calculated using the following formulas: PPV = TP/(TP + FP) × 100, estimating the probability that a positive test result indicates the presence of the disease, and NPV = TN/(TN + FN) × 100, estimating the probability that a negative test result indicates the absence of the disease. The assay cut-off point was determined by analyzing the ROC curve (area under the curve) of the test readings obtained from samples of both sick and healthy animals.

The repeatability and reproducibility of the assay were determined using the coefficient of variation (CV: standard deviation/mean × 100%). Values below 15% were considered ideal. The detection limit was established by adding three times the standard deviation to the mean absorbance of the blank (DL = mean + 3SD). In the repeatability test, twenty repetitions were performed with two serum samples known to be positive for SAAeq and two serum samples known to be negative for SAAeq. For the reproducibility test, the same samples were evaluated in triplicate in three separate assays, with a 1-week interval between each. Different reagents were used in each assay.

To quantify SAAeq, a standard curve was constructed using linear regression parameters based on the serial dilution of rSAAeq with an initial concentration of 150 mg/L. Each dilution was tested in duplicate and the average absorbance values were used to construct the standard curve.

### 2.8. Statistical Methods

The arithmetic means, standard deviations, and intra- and inter-assay coefficients of variation were calculated using descriptive statistical procedures. Then, to determine the cut-off, sensitivity, and specificity of the test, the data were analyzed using GraphPad Prism 8.0 software. This analysis was performed utilizing the optical density values of the positive and negative serological samples for SAAeq.

## 3. Results

**Figure 1:**
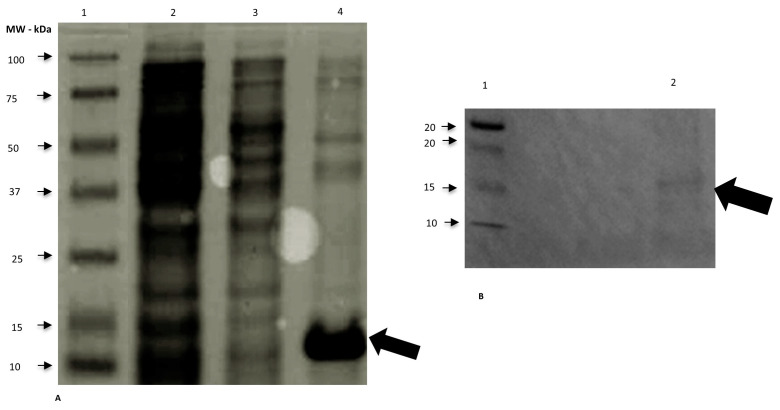
Confirmation of rSAAeq expression in *E. coli* BL21-CodonPlus by 15% SDS-PAGE and Western blotting at 37 °C, induced by 0.4 mM IPTG/4 h. (**A**) Line 1 —MW—Bio-Rad molecular weight marker; Line 2—uninduced insoluble fraction; Line 3—induced soluble fraction; Line 4—Purified rSAAeq—presence of rSAAeq suggested at approximately 14 kDa. (**B**) Western blotting with anti-His antibody produced in mouse—presence of rSAAeq suggested at approximately 14 kDa.

We expressed the rSAAeq in the soluble fraction of competent *E. coli* BL21-CodonPlus colonies at 37 °C for 4 h and at all tested concentrations of IPTG (0.4, 0.8, and 1 mM). We observed protein expression and purification using 15% SDS-PAGE gel and characterized them through Western blotting (Figure 1). We successfully performed protein dialysis.

**Figure 2:**
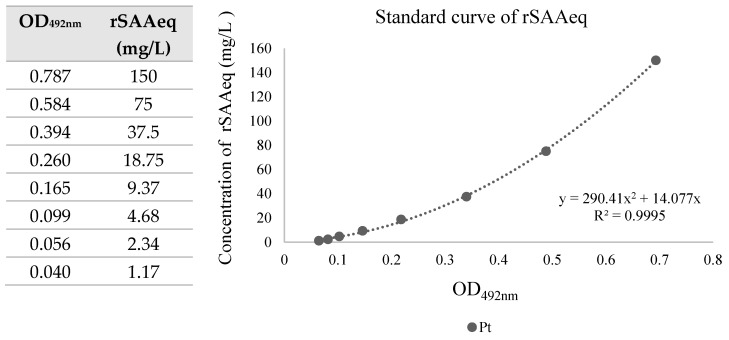
Standard curve of rSAAeq for quantification of equine serum amyloid A. SAA, serum amyloid A; OD, optical density.

To standardize the test, we sensitized ELISA plates with the optimal concentration of capture antibodies and purified rSAAeq, determined from previous dilutions. We used these for constructing the standard curve. We constructed the standard curve with anti-SAAeq at dilutions of 1:100 for anti-SAAeq1 and 1:80 for anti-SAAeq2. From serial dilutions of rSAAeq with an initial concentration of 150 mg/L and a final concentration of 1.17 mg/L, we obtained an average OD_492_nm of 0.787 and 0.040, respectively, with a high correlation coefficient of R^2^ = 0.9995 (Figure 2).

**Figure 3:**
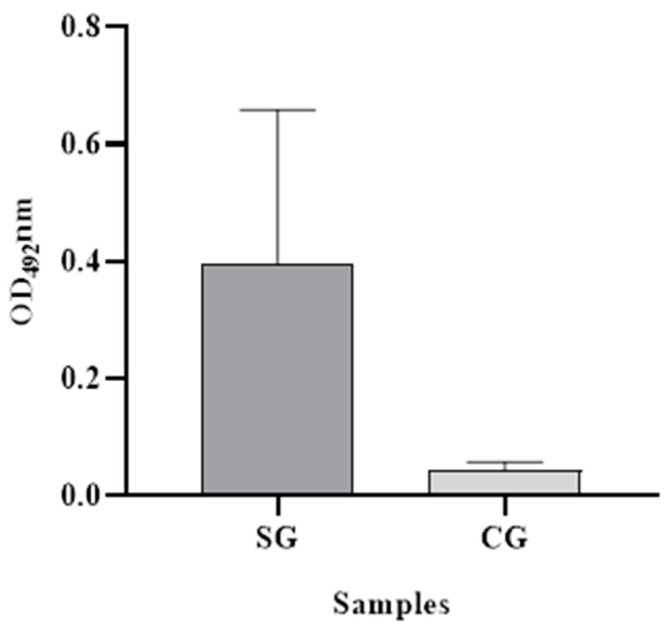
Mean and standard deviation of the absorbance of positive and negative serological samples for SAAeq. SG (animals with acute inflammatory disease) and CG (healthy animals).

The ability of the assay to diagnose SAAeq in serum samples from animals with acute inflammatory disease (positive) and animals with baseline protein concentrations (negative) was evaluated in the field. The dilution of the samples that demonstrated the best interaction with the standardized antibodies was determined to be 1:50. The positive group exhibited a mean OD_492_nm value of 0.398 (SD = 0.262), while the negative group exhibited a mean value of 0.044 (SD = 0.013) (Figure 3). Additionally, the cut-off point determination was established at 0.06 OD_492_nm, with sensitivity and specificity values clearly reported.

**Figure 4:**
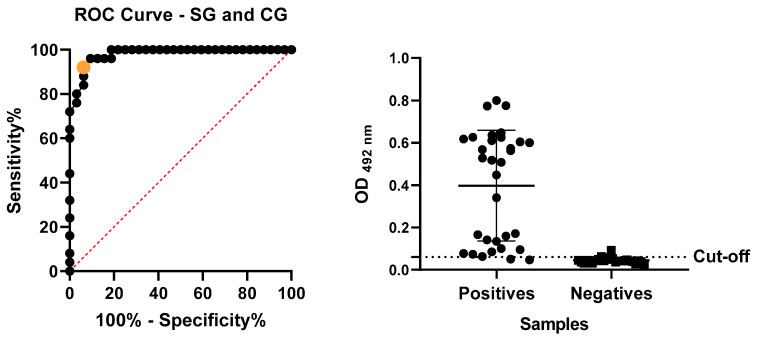
Curve ROC—analysis of the test readings performed with samples from sick and healthy animals to determine the cut-off point. Orange dot: 94% sensitivity and 92% specificity. Cut-off 0.06.

We evaluated the absorbance readings of the SAA-ELISA assay, performed with positive and negative samples, by analyzing the ROC curve (Figure 4) to determine the cut-off. This analysis established the cut-off at 0.06. We considered the mean of each tested sample with an OD_492_nm value higher than 0.06 to be positive and samples below this value were considered negative. The obtained performance rates, including sensitivity, specificity, accuracy, PPV, and NPV (Table 1), were explicitly calculated based on these threshold values.

Inter- and intra-assay CVs were observed to range from 6.1% to 9.6% (corresponding to OD_492_nm values between 0.790 and 0.821 for sick samples and 0.052 and 0.059 for healthy samples) and from 7.46% to 9.63% (OD_492_nm values between 0.764 and 0.717 for sick samples and 0.086 and 0.088 for healthy samples), respectively (Table 2). These measurements were performed on samples from both sick and healthy animals. The detection limit was obtained as 0.067 OD_492_nm, which falls within the range of the assay’s reproducibility, ensuring reliable detection of low SAAeq concentrations.

**Figure 5:**
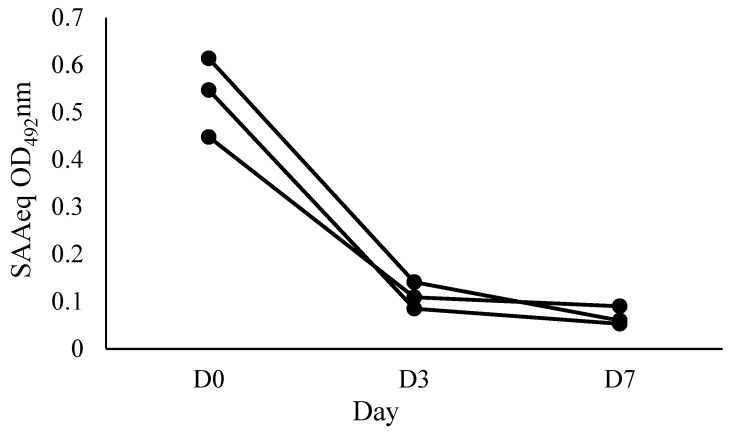
The applicability of SAA-ELISA in monitoring the clinical evolution of horses with acute inflammatory disease (dental fistula, hemoparasitosis, and penile laceration) evaluated individually at three time points (D0—arrival at the hospital; D3—3 days after the start of treatment; and D7—7 days after the start of treatment). Each line represents an animal.

The applicability of SAA-ELISA in monitoring the clinical evolution of patients with acute inflammatory disease, by observing the kinetics of SAAeq, was evaluated at three time points: D0 (arrival at the hospital), D3 (3 days after the start of treatment), and D7 (7 days after the start of treatment) (Figure 5).

## 4. Discussion

We widely utilize the system with bacterial host cells, particularly *E. coli*, for producing heterologous proteins with good results, even though it has some limitations [13]. The choice of the most suitable system depends on our understanding of the desired protein and the production objective. We considered the production of the SAAeq protein using this system as satisfactory. The visualization of a band of interest at the height of 14 kDa suggested the presence of SAAeq, as described by [3]. These authors assert that the denatured form of this high-density lipoprotein protein might weigh around 9 to 14 kDa. However, we encountered difficulty purifying the heterologous protein, corroborating [23]. Additional resources, such as the insertion of fusion proteins into a new plasmid, could improve the expression, folding, and stability of the target protein [24].

We use ELISA, a highly sensitive immunological test, to diagnose and monitor diseases through the antigen-antibody reaction [18,25]. This technique has several types, including one for equine SAA detection. However, the lack of a readily available national product in the Brazilian market makes the test expensive to import, limiting its application in both equine clinical routine and research development.

To develop a highly sensitive and specific test with a lower chance of cross-reactivity, we employed the sandwich ELISA technique in this study. This approach involved using two polyclonal antibodies against SAAeq produced in different species. This strategy enabled the effective capture of the target protein (SAAeq), leading to more consistent results.

Choosing species-specific antibodies also ensured their higher affinity for binding with the native protein, minimizing potential interference from other proteins and enhancing the test’s accuracy. This strategy resulted in a more reliable and accurate test, which is critical in disease diagnosis and monitoring. Additionally, the specificity of the binding allowed us to quantify the antigen, leading to greater result reproducibility [7].

We were satisfied with the results obtained by the SAA-ELISA. The OD_492_nm values observed in the group of sick animals differed significantly from those in the group of healthy animals. This difference allowed us to precisely differentiate between the two groups with high sensitivity and specificity.

While no result curve is perfect, the parameter we adopted for selecting positive samples in this study was stringent, using clinical examination and mass spectrometry as the gold standard. This criterion allowed us to select samples with high concentrations of SAAeq, at the peak of the inflammatory process. This enabled the test to precisely identify sick animals.

In equine clinical practice, early identification of an inflammatory process is critical [4]. Establishing an appropriate cut-off point for a diagnostic test is essential for determining its sensitivity and specificity, considering the disease process’s duration and the agent involved [17]. In this study, ELISA has proven satisfactory for this purpose.

Several factors can affect the sensitivity and specificity of early inflammatory markers like SAA, including the phase and intensity of the inflammatory process and the agent involved. Setting a cut-off point above the normal value can enhance the test’s sensitivity while maintaining specificity [19]. Currently, human SAA measurement tests are used to validate SAA measurements in horses and other species and the sensitivity and specificity of human SAA tests in horses vary depending on the method used. The ELISA for equine SAA showed high reliability, with a sensitivity of 0.93 and specificity of 0.77 at a cut-off of 23.95 mg/L [26,27]. On the other hand, a turbidimetric immunoassay (TIA) developed for humans showed intra-assay imprecision ranging from 1.6% to 24.4% and inter-assay imprecision from 4.6% to 33.2% [17]. Corroborating our findings, studies in cats have demonstrated high sensitivity and specificity (93% and 99%, respectively) using these tests [28]. However, in cattle, sensitivity and specificity were 86.7% and 75%, respectively [29]. In dogs, low sensitivity and high specificity were reported (43.2% and 92.3%, respectively) [30]. Interpreting diagnostic test results depends on the test’s purpose and the requesting professional’s technical expertise.

We found that the precision measurements evaluated in this test were consistent with ideal values, ensuring its ability to produce accurate results. In the inter-assay, the mean coefficients of variation (CV) for positive and negative samples were 6.1% and 9.6%, respectively, while in the intra-assay, they were 7.46% and 9.63%, respectively. These values were consistently lower than those reported for previously validated tests for SAAeq measurement: 24.4% and 33.2% for inter-assay and 5.7–12% for intra-assay [17] and 7.8–13.3% for intra-assay [6]. Similar results to this study were described [20], who reported CVs in the inter-assay of 9–5.5% and in the intra-assay of 11.7–4.6% at low and high concentrations, respectively. Lower CV values were also reported in the inter-assay (11.6–3%) and intra-assay (2.1–3.2%) with intermediate and high SAAeq concentrations, and in the inter-assay (3–5.2%) and intra-assay (6.8–9.6%) with low, intermediate, and high concentrations [19,31]. Although there is a high rate of variation in the CV that can occur due to batch differences [21], smaller variations are considered ideal for a diagnostic test. Therefore, the results of this test are reliable and indicate that the developed test has good precision, which can be an important factor for its clinical application.

We evaluated the precision of the test and found that the measures remained within the ideal range for a diagnostic test. These measures guarantee the test’s ability to produce consistent results. Repeatability aims to determine whether the developed test can maintain the results obtained with the same samples tested multiple times under identical conditions (same day, operator, equipment, and samples). Reproducibility, on the other hand, assesses the test’s ability to maintain consistent results even when different conditions are used to analyze the same samples [32].

For equine medicine, SAA is the most important acute-phase protein. Its baseline levels are incredibly low, almost undetectable, and it has a short half-life. Therefore, when tissue injury occurs, SAA levels rapidly rise within hours, peaking around 24 h. Once the stimulus ceases, the concentration swiftly decreases [8,17]. The test’s performance was also satisfactory in this regard, demonstrating the ability to track SAAeq kinetics in field samples from horses naturally affected by acute inflammatory disease. Similar findings were described by [17] when measuring SAA using an immunoturbidimetric test in horses before and after castration. This characteristic allows SAA-ELISA to be used for monitoring the clinical evolution of patients. This enables us to evaluate the response to the instituted therapy and establish a prognosis.

Despite the development of various SAA measurement methods over the years, including electroimmunoassay [18], simple radial immunodiffusion [33], ELISA [22], and immunoturbidimetric assays [34], accurately quantifying this protein remains a challenge. Currently, no single method reigns supreme as the gold standard. To measure the protein amount in a sample, we can utilize a standard curve correlating known concentrations of a specific protein with OD_492_nm values. We constructed a standard curve using purified rSAAeq, achieving a correlation coefficient of R^2^ = 0.9521. This indicates linearity between the absorbance values and the concentration of the analyzed protein. While the SAA-ELISA is a quantitative test, further optimization is recommended to obtain more accurate results and define its measurement capability.

Currently, veterinary practitioners use the LZ-SAA and VET-SAA immunochromatographic tests (quantitative) and the POC (qualitative lateral flow) to measure SAAeq [35], However, comparisons between these methods have revealed variations in protein quantification due to differences in antibody affinity towards different protein isoforms [19].

The SAA-ELISA test demonstrated good performance in detecting SAAeq. It achieved a sensitivity of 94%, a specificity of 92%, and an overall accuracy of 93%. The positive and negative predictive values were both 94%. The precision results showed a coefficient of variation (CV) of 6.1% for the patient group and 9.6% for the control group in the inter-assay and a CV of 7.46% for the patient group and 9.63% for the control group in the intra-assay. While further optimization is needed to improve accuracy, the obtained data suggest that SAA-ELISA is a highly sensitive and specific tool with potential utility in detecting SAAeq in equine serum samples.

## Figures and Tables

**Table 1:** Performance rates evaluated in SAA-ELISA for the diagnosis of SAAeq.

True Positives	n = 32
False positives	n = 2
True negatives	n = 2
False negatives	n = 25
Sensitivity	94%
Specificity	92%
Accuracy	93%
PPV	94%
NPV	92%

PPV, positive predictive value; NPV, negative predictive value.

**Table 2:** Coefficients of variation for inter- and intra-assay evaluation through OD_492_nm observed in the SAA-ELISA based on anti-SAAeq polyclonal antibodies.

		SAA (OD_492nm_)			
Precision Measurements		Sample	Mean	SD	CV (%)
Intra-assay	Sick	1	0.764	0.055	7.24
		2	0.717	0.036	4.95
	Healthy	1	0.086	0.009	10.16
		2	0.088	0.008	9.03
Inter-assay	Sick	1	0.790	0.006	7.75
		2	0.821	0.058	7.16
	Healthy	1	0.052	0.005	10.66
		2	0.059	0.005	8.6

SAA, serum amyloid A; OD, optical density; SD, standard deviation, CV, coefficient of variation.

## Data Availability

The data presented in this study are available from the corresponding author upon request.
